# ACOT7 protects epidermal stem cells against lipid peroxidation

**DOI:** 10.1007/s11626-022-00703-9

**Published:** 2022-08-29

**Authors:** Guang Zhang, Jiaxu Ma, Zhenjie Wu, Guoqi Cao, Chunyan Liu, Ru Song, Rui Sun, Aoyu Chen, Yibing Wang, Siyuan Yin

**Affiliations:** 1 Department of Plastic Surgery, Shandong Provincial Qianfoshan Hospital, Cheeloo College of Medicine, Shandong University, Jinan, Shandong 250012 People’s Republic of China; 2 Jinan Clinical Research Center for Tissue Engineering Skin Regeneration and Wound Repair, The First Affiliated Hospital of Shandong First Medical University & Shandong Provincial Qianfoshan Hospital, Jinan, 250014 People’s Republic of China; 3 Department of Plastic Surgery, The First Affiliated Hospital of Shandong First Medical University & Shandong Provincial Qianfoshan Hospital, Jinan, 250014 People’s Republic of China

**Keywords:** Transferrin, ACOT7, Epidermal stem cells, Lipid peroxidation

## Abstract

Epidermal stem cells (ESCs) are critical for skin regeneration and repair. Previous studies have shown that ESCs are susceptible to oxidative stress, which in turn leads to lipid peroxidation and affects skin repair. Our study aims to explore how ESCs resist lipid peroxidation. By performing proteomics analysis, we found that the expression of Acyl-CoA thioesterase 7 (ACOT7) was positively correlated with the concentration of transferrin. Overexpression adenovirus vectors of ACOT7 were constructed and transfected into ESCs. Levels of lipid peroxidation by flow cytometry, cell viabilities, and MDA levels were measured. The results revealed that ACOT7 could inhibit lipid peroxidation, reduce the level of malondialdehyde (MDA), and improve the survival rate of ESCs induced by H_2_O_2_, Erastin, and RSL3. Our data suggest that ACOT7 has an effect on protecting ESCs against iron-dependent lipid peroxidation.

## Introduction

The skin is the largest organ of the human body, which constantly renews itself to maintain its barrier function (Hennig *et al*. 2020). Being directly exposed to the environment, the skin is easily affected by ultraviolet radiation, pathogen invasion, and other external threats (Yin *et al*. 2019). These factors can cause oxidative stress, which in turn affects the function of skin cells. The epidermis, the outermost layer of the skin, is rich in lipids, proteins, and DNA, so it is susceptible to oxidative stress. Epidermal damage can lead to skin aging, poor wound healing, and even skin diseases (Klaunig *et al*. 2010; An *et al*. 2013). ESCs are a type of cells located in the basal part of the epidermis, which can differentiate into mature keratinocytes that have an indispensable role during skin healing after trauma (Liao *et al*. 2014). However, ESCs are easily affected by oxidative stress, which may affect their ability to proliferate and differentiate, thus delaying the wound healing process (Ud-Din *et al*. 2021).

Iron is a catalyst of oxidative stress. It can bind to different ligands and has the property of electron transfer (Wang *et al*. 2019). Excessive amounts of intracellular iron may destroy redox homeostasis through the Fenton reaction and catalyze reactive oxygen species (ROS) production, inducing the destruction of macromolecular substances, such as DNA, RNA, protein, and lipid (Fibach 2019). ROS then interacts with polyunsaturated fatty acids on the biomembrane, resulting in cellular structure and function changes, a process also known as lipid peroxidation, which then leads to cell death, i.e., ferroptosis (Tomita *et al*. 2019). Previous studies suggested that oxidative stress increases with overload iron, which may harm cell functions. This process may be ameliorated by chelating excess intracellular free iron through deferoxamine (Holden and Nair 2019). In addition, cells may upregulate the expression of some genes, such as ferritin, to relieve the effect. According to previous studies, in keratinocytes and fibroblasts, overexpressed ferritin caused by iron load may significantly inhibit the procedure of lipid peroxidation (Giordani *et al*. 2000; Zhang *et al*. 2021). However, the impact of iron load on ESCs needs to be further explored.

ACOT7 is an enzyme that catalyzes the hydrolysis procedure from acyl-CoA to free fatty acid and coenzyme A. In our study, we found that ACOT7 reduces the level of iron-dependent lipid peroxidation. The aim of the present study was to further explore the role of ACOT7 in inhibiting the procedure of lipid peroxidation in ESCs cultured in vitro.

## Materials and methods

### Animals

All animal studies (including the mouse euthanasia procedure) were done in compliance with Shandong University institutional animal care regulations and conducted according to the ARRIVE guidelines and the U.K. Animals (Scientific Procedures) Act 1986 guidelines. A total of 20 specific pathogen-free (SPF) wild-type male C57BL/6 J newborn mice were purchased from the experimental animal center of Shandong University (Permit number: SCXK Lu 20,190,001). All the animals were housed in an environment with a temperature of 22 ± 1°C, relative humidity of 50 ± 1%, and a light/dark cycle of 12/12 h.

### Isolation, culture, and identification of ESCs

Processes of isolation, culture, and identification of ESCs were conducted according to previous studies (Zhang *et al*. 2018a, b, 2020). Briefly, 20 mice were used for this experiment. Skin tissues were collected and digested at + 4°C overnight in 0.5% Dispase II (Sigma, St. Louis, MO). Then, the epidermis was dissected from the dermis and digested in 0.25% trypsin (Gibco, Crand Island, NY) at + 37°C for 7 min; the process of digestion was terminated by adding RMPI 1640 (Thermo Fisher Scientific, Shanghai, China) and 10% FBS (Thermo Fisher Scientific). Samples were then filtrated and centrifuged to obtain cells, which were resuspended in a CnT-Prime culture medium (Celltec, Bern, Switzerland).

Cells were incubated in culture bottles containing type IV collagen (Sigma) at the density of 5 × 10^5^–1 × 10^6^/ml, and they were placed in normoxic incubators containing 5% CO2 at + 37°C for approximately 30 min. After cell adherence, the culture solution was discarded, and cells were gently rinsed with phosphate-buffered saline (PBS) three times. Cells were incubated in a fresh medium in normoxic incubators, and the culture solution was conducted every other day.

Flow cytometry, Western blot analysis, and immunofluorescence analysis were used to identify ESCs. Cytokeratin 14 (CK14), cytokeratin 15 (CK15), p63, and cytokeratin 10 (CK10) were used to differentiate ESCs from keratinocytes.

All experiments were performed with mycoplasma-free cells.

### TMT quantitative proteomic analysis

ESCs were cultured in the medium containing 100 µg/ml holo-transferrin (Sigma) (experimental group) or without holo-transferrin (control group) for 2 d. Three samples from each group were used for TMT quantitative proteomic analysis. The TMT quantitative proteomic analysis was performed by Shanghai Applied Protein Technology Co., Ltd. (Project’s number: P20200801875).

### Adenovirus transfection

After reaching a 70% confluency, the adenovirus carrying *Acot7* (with Flag tag) and the vector adenovirus (without Flag tag) were transfected into ESCs. The multiplicity of infection (MOI) was determined to be 10. The ESCs were divided into the following three groups: (a) experimental group (ESCs were transfected by the adenovirus carrying *Acot7*); (b) control group (ESCs were transfected by the vector adenovirus); (c) the blank group (ESCs did not receive adenovirus transfection). The adenoviruses were provided by Shanghai Genechem Co., Ltd, Shanghai, China. The culture mediums were changed after the 10-h period of transfection. On the second day, Western blot analysis and immunofluorescence analysis were applied to investigate the transfection efficiency.

### Detection of cell proliferation

Cell proliferation was analyzed using an IncuCyte S3 Living-Cell Analysis System (Sartorius, Gottingen, Germany). Briefly, ESCs were cultured in 96-well plates at the density of 5 × 10^3^ cells/plate, and then incubated with CnT-Prime culture medium at 37°C and 5% CO2 for 48 h. By calculating the area confluence, which was normalized to 0 h, then the cell proliferation curves were drawn.

### Detection of cell apoptosis

The Annexin V-PE/7-AAD apoptosis analysis kits (BD Biosciences, San Jose, CA) detected apoptosis (Meng *et al*. 2018). After centrifugation and washing with PBS, ESCs were resuspended using 1 × binding buffer at a density of 1 × 10^6^ cells/ml, and then incubated with PE-conjugated Annexin V and 5 µl of 7-AAD for 15 min at room temperature (25°C) in the dark. Afterwards, samples were detected by CytoFLEX flow cytometer (Beckman Coulter, Indianapolis, IN). Early apoptotic, late apoptotic, and dead cells can be distinguished on the basis of a double-labeling for Annexin V-PE and 7-AAD.

### Detection of cell viability

The impacts of H_2_O_2_, Erastin, and RSL3 on the cell viability of ESCs were measured by the Cell Counting Kit-8 (CCK-8) (Dojindo, Kumamoto, Japan) method. Cells were inoculated in 96-well plates, and 500 µM H2O2 (Sigma), 20 µM Erastin (MedChemExpress, Shanghai, China), and 5 µM RSL3 (MedChemExpress) were added into plates. After 6 h, the culture solution was discarded. Cells were then incubated in 100 μl fresh culture solution and 10 μl CCK-8 at 37°C for 2 h. Next, the absorbance of the solution at 450 nm was measured by Spark (Tecan, Salzburg, Austria) to calculate the cell viability of ESCs.

### Detection of the relative levels of lipid peroxidation and Fe.^2+^ by flow cytometry

The relative intracellular levels of lipid peroxidation and Fe^2+^ were measured by the C11-BODIPY lipid probe (Invitrogen, Carlsbad, CA) and FerroOrange (Dojindo). The cells were gently rinsed with PBS three times, and the adherent cells were digested with trypsin. After digestion, centrifugation was conducted to collect cell pellets, which were resuspended with 100-µl living cell image solution (Thermo Fisher Scientific), and incubated with 0.1 µl C11-BODIPY (Invitrogen, 1:1000) and 0.2 µl FerroOrange (Dojindo, 1:500) in the dark for 30 min. Next, to blow and mix the cells, a 400-µl living cell image solution was added. The wavelength of FerroOrange excitation light was 543 nm, and the wavelength of emission light was 580 nm. Oxidation of the polyunsaturated butadienyl portion of the C11-BODIPY resulted in a shift of the fluorescence emission peak from ~ 590 to ~ 510 nm. The relative levels of lipid peroxidation and Fe^2+^ were measured by flow cytometry, which was quantified by the FITC/PE value and the PE value, respectively (Cheloni and Slaveykova 2013; Mei *et al*. 2020).

### Detection of MDA levels

The intracellular level of MDA was determined according to the instructions of lipid peroxidation (MDA) assay kit (Abcam, Cambridge, UK). In the lipid peroxidation assay protocol, the MDA in the sample was reacted with thiobarbituric acid (TBA) to generate an MDA-TBA adduct. The MDA-TBA adduct was easily quantified colorimetrically (OD = 532 nm) (Wang *et al*. 2021).

### Protein extraction and Western blot

Western blot analysis was performed in order to measure the expression of proteins. The cell lysate was attained with RIPA lysate (Thermo Fisher Scientific) and the protein concentration of the samples was measured with BCA Protein Assay Kit (Thermo Fisher Scientific). Samples (approximately 40 µg) were separated by 10% SDS-PAGE and transferred to PVDF membranes. The membranes were blocked in 5% non-fat milk for 2 h before being incubated with primary antibodies at 37°C overnight. The primary antibodies including anti-CK10 (1:1000, Abcam, ab76318), anti-CK14 (1:1000, Abcam, ab181595), anti-CK15 (1:1000, Abcam, ab52816), anti-p63 (1:1000, Abcam, ab124762), anti-GAPDH (1:1000, Cell Signaling Technology, #5174, Boston, MA), anti-ACOT7 (1:5000, Proteintech Group, 15,972–1-AP), anti-FLAG (1:500, Sigma, F7425), anti-FTH (1:1000, Invitrogen, 701,934), and anti-FTL (1:1000, Invitrogen, MA5-32,755). The membranes were then rinsed with TBST and incubated with the HRP-conjugated secondary antibody (1:5000, Cell Signaling Technology, #7074) at room temperature for 1 h. The iBright imaging system (Invitrogen) was applied to obtain images, and the ImageJ software was used to quantify the expression of proteins.

### Immunofluorescence

Cell samples were fixed in 4% paraformaldehyde for 15 min at room temperature and washed three times in PBS, permeabilized 0.5% TritonX-100/DPBS (Invitrogen) for 10 min, and blocked with blocking solution for 1 h. After overnight incubation at 4℃ with primary antibodies, cell samples were incubated with secondary antibodies for 1 h. Finally, the cell nuclei were stained with DAPI. The antibody information has been described before. Cell immunofluorescence images were captured by an A1R confocal microscope (Nikon, Belmont, CA).

### Statistical analysis

We used the GraphPad Prism version 8.0.1 for statistical analysis, and the quantitative data were expressed as mean ± standard deviation. All data were attained from at least three independent experiments. Group differences were explored by using the unpaired *t*-test, where *P* < 0.05 represented statistical significance. Asterisks represent significance between conditions for each group. *P*-values are labeled as *P < 0.05, **P < 0.01, and ***P < 0.001.

## Results

### ESCs showed high expression of CK14, CK15, and p63

**Figure 1.**
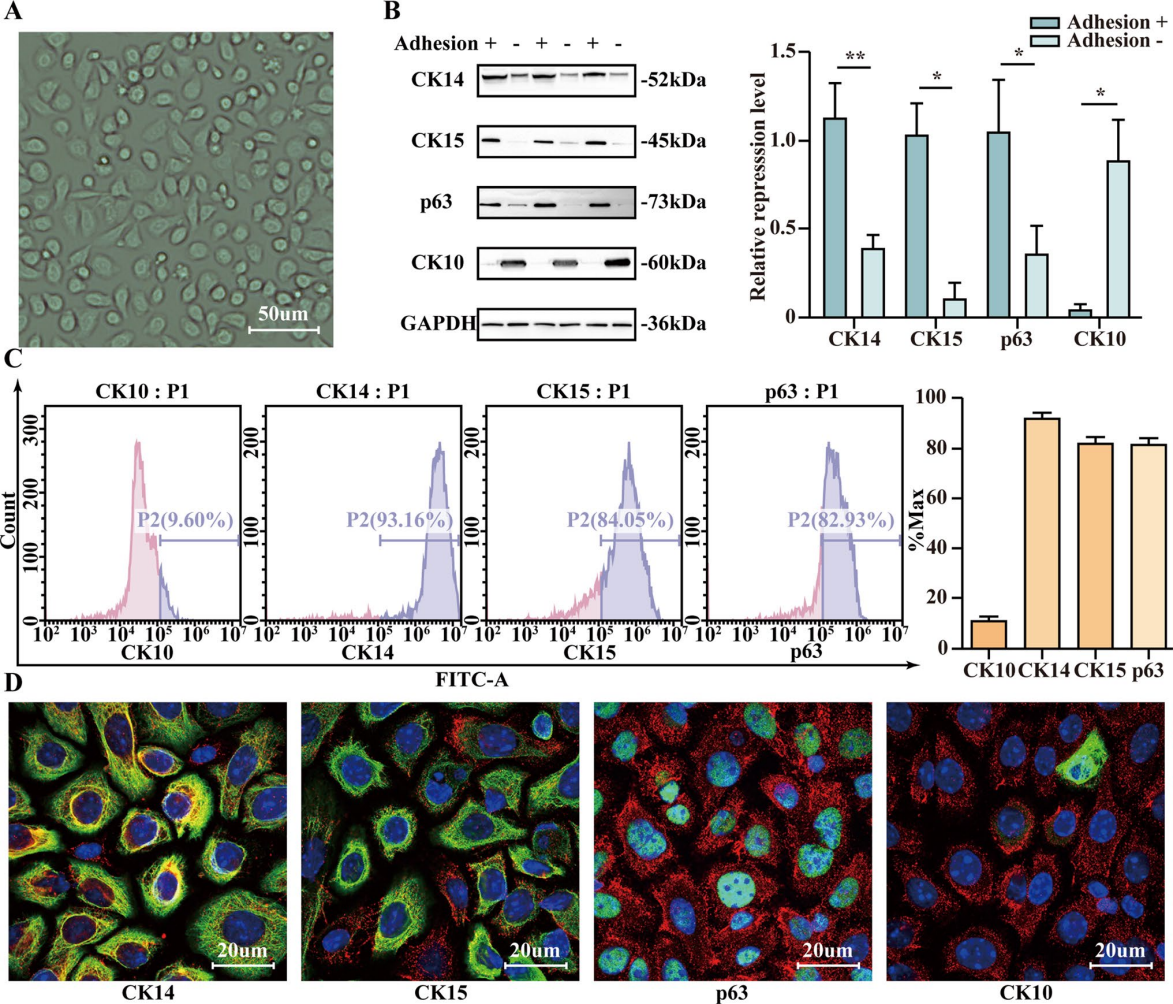
ESCs showed high expression of CK14, CK15, and p63. (*A*) ESCs cultured for 2 d. *Scale bar* = 50 µm. (*B*) WB and quantification showed CK14, CK15, p63, and CK10 expression levels between adherent and non-adherent cells. (*C*) Flow cytometry and quantification of positive rates of obtained cells expressing CK14, CK15, p63, and CK10. (*D*) Immunofluorescence identification of obtained cells expressing CK14, CK15, p63, and CK10. *Scale bar* = 20 µm. Statistics: Data were obtained in triplicate experiments and are shown as the mean ± SD; unpaired *t*-test. *P < 0.05, **P < 0.01.

After 2 days of culture, ESCs were observed under a light microscope (Fig. 1*A*). CK14, CK15, and p63 are markers of ESCs and CK10 is the marker of keratinocytes (Ghadially 2012). Next, Western blot analysis was performed to compare markers expressed on adherent and non-adherent cells. The expression of CK14, CK15, and p63 was significantly higher in the adherent cells than that in the non-adherent cells, and the expression of CK10 was significantly lower in the adherent cells than that in the non-adherent cells (Fig. 1*B*). Furthermore, flow cytometry suggested that the positive rates of obtained cells expressing CK14, CK15, and p63 were more than 80%, while the positive rate of CK10 was less than 10% (Fig. 1*C*). Similar results were observed by immunofluorescence, which suggested that these cells were ESCs (Fig. 1*D*).

### The expression of ACOT7 increases with the rise of iron load in ESCs

**Figure 2.**
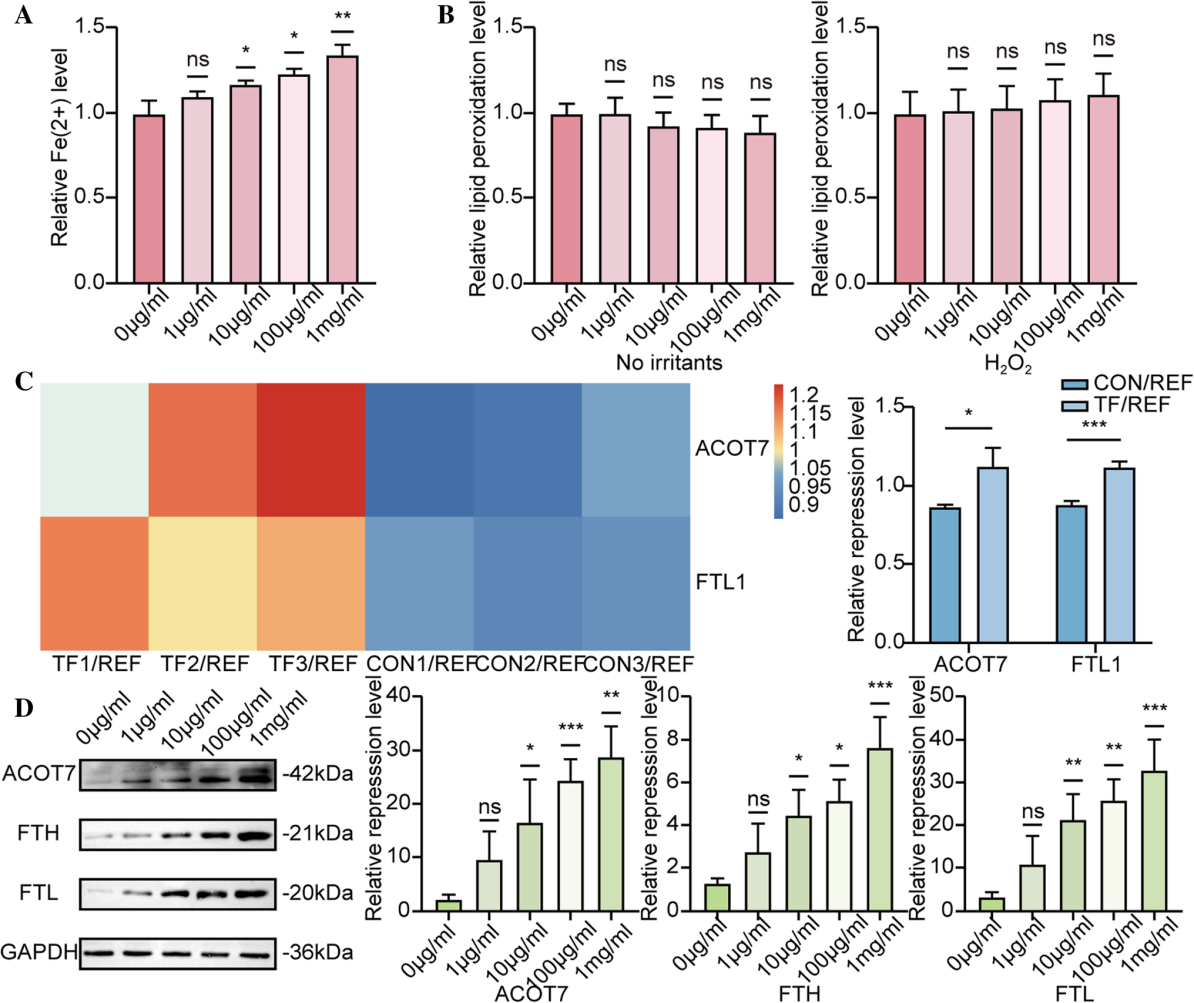
The expression of ACOT7 increases with the rise of iron load in ESCs. (*A*) Flow cytometry quantification of cellular Fe^2+^ level in the medium with 0, 1, 10, 100, or 1000 µg/ml holo-transferrin. (*B*) Flow cytometry quantification of cellular lipid peroxidation level with no irritants and 500 µM H_2_O_2_. (*C*) ESCs cultured in the medium with 100 μg/ml holo-transferrin and no holo-transferrin; ACOT7 and FTL1 showed differences through TMT quantitative proteomic analysis. (*D*) WB and quantification showed ACOT7, FTL, and FTH expression levels in the medium with 0, 1, 10, 100, or 1000 µg/ml holo-transferrin. Statistics: Data were obtained in triplicate experiments and are shown as the mean ± SD; unpaired *t*-test. *P < 0.05, **P < 0.01, ***P < 0.001. *ns*, not significant.

Next, ESCs were cultured in the medium with 0, 1, 10, 100, or 1000 µg/ml holo-transferrin. Flow cytometry suggested that with the increase of the concentration of holo-transferrin, there was a significant rise in the level of intracellular Fe^2+^ (Fig. 2*A*), while no differences were observed in the level of lipid peroxidation. After the ESCs were stimulated with 500 µM H_2_O_2_ for 6 h, no differences in the level of lipid peroxidation were observed (Fig. 2*B*). Because of the Fenton reaction, we suspected that some genes were upregulated to maintain a stable level of lipid peroxidation. Consequently, we found significant differences in the expression of some proteins, including ACOT7 and Ferritin Light Chain1 (FTL1), through TMT quantitative proteomic analysis (Fig. 2*C*), which was further confirmed by Western blot (WB). WB analysis suggested that ACOT7, Ferritin Heavy Chain (FTH), and Ferritin Light Chain (FTL) upregulation was positively correlated with the concentration of transferrin (Fig. 2*D*).

### The overexpression of ACOT7 has no impact on the proliferation and apoptosis of ESCs

**Figure 3.**
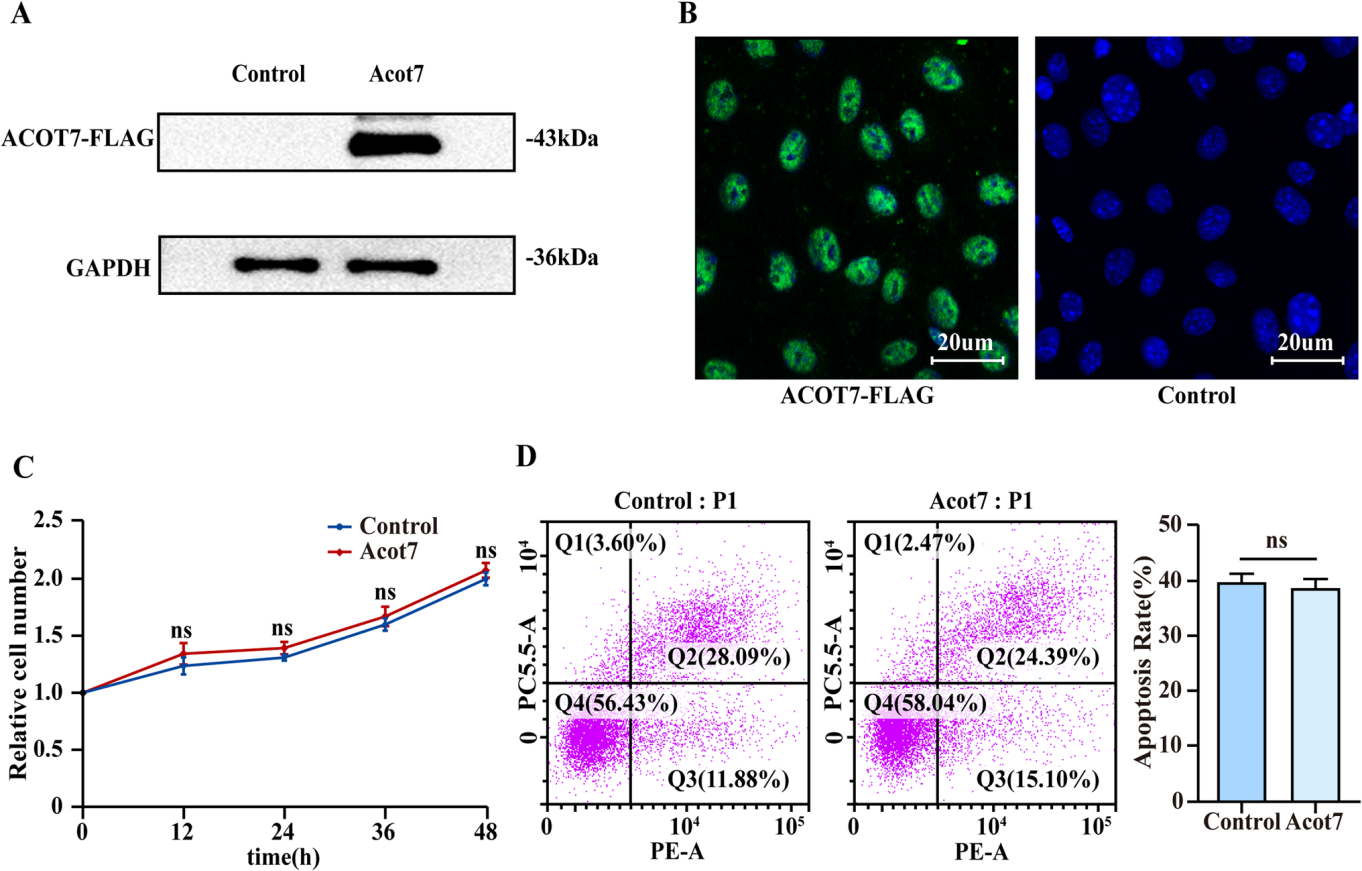
The overexpression of ACOT7 has no impact on the proliferation and apoptosis of ESCs. (*A, B*) WB and immunofluorescence showed Flag expression levels between experimental group (ESCs were transfected by the adenovirus carrying *Acot7*) and the control group (ESCs were transfected by the vector adenovirus). *Scale bar* = 20 µm. (*C*) IncuCyte S3 Living-Cell Analysis System measured cell proliferation. (*D*) Flow cytometry detected cell apoptosis between experimental group and the control group. Statistics: Data were obtained in triplicate experiments and are shown as the mean ± SD; unpaired *t*-test. *ns*, not significant.

The experimental group (ESCs were transfected by the adenovirus carrying *Acot7*) and the control group (ESCs were transfected by the vector adenovirus) were differentiated using the Flag tag. Western blot analysis and immunofluorescence analysis suggested that ESCs in the experimental group expressed a high Flag level compared to the control group (Fig. 3*A* and *B*). The results indicated the efficiency of virus transfection in the present study. Moreover, the cell proliferation experiment and flow cytometry result showed no differences in the cell proliferation curve and the apoptosis ratio between the two groups (Fig. 3*C* and *D*).

### The overexpression of ACOT7 decreases the levels of MDA and lipid peroxidation

**Figure 4.**
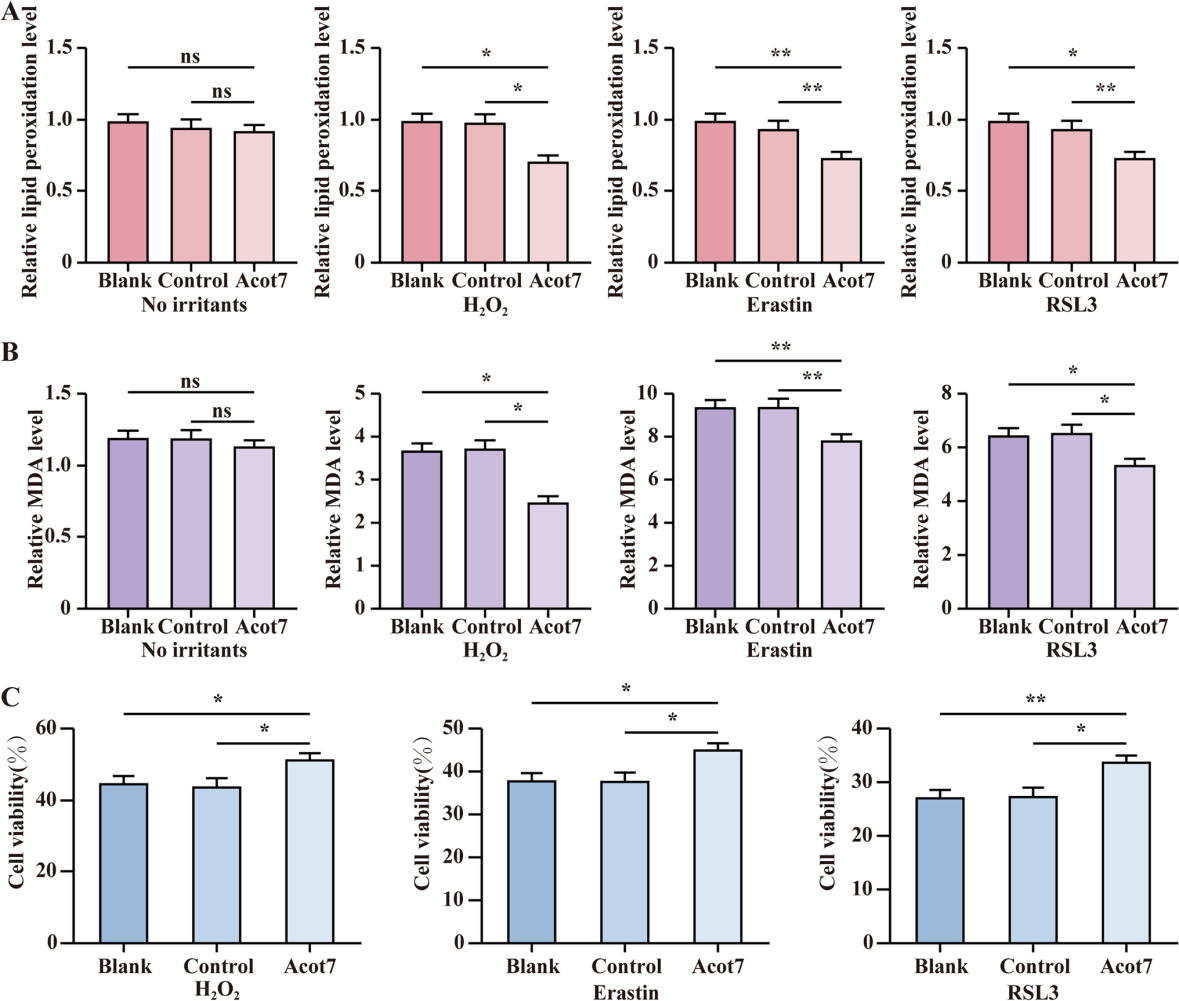
The overexpression of ACOT7 decreases the levels of MDA and lipid peroxidation. (*A*) Flow cytometry detected cellular lipid peroxidation levels with no irritants, H_2_O_2_, Erastin, and RSL3. (*B*) MDA assay showed MDA relative levels with no irritants, H_2_O_2_, Erastin, and RSL3. (*C*) CCK-8 experiment measured cell viability with H_2_O_2_, Erastin, and RSL3. Statistics: Data were obtained in triplicate experiments and are shown as the mean ± SD; unpaired *t*-test. *P < 0.05, **P < 0.01. *ns*, not significant.

To verify the function of ACOT7, we divided the cells into the following three groups: the experimental group, the control group, and the blank group. There were no differences in the level of lipid peroxidation measured by flow cytometry without *oxidative stress*. After being incubated with H_2_O_2_, Erastin, and RSL3 for 6 h, the lipid peroxidation level was significantly lower in the experimental group than that in the control and blank groups (Fig. 4*A*). We also found that under the condition of the same oxidative stress, the MDA level was significantly lower in the experimental group than that in the control and blank groups (Fig. 4*B*). Besides, the result of the CCK-8 experiment showed that the cell viability of ESCs was significantly higher in the experimental group than that in the control and blank groups (Fig. 4*C*).

## Discussion

Iron plays a key role in oxidative stress and light-induced skin damage. It is essential for cells to regulate the balance of oxidative stress (Wang *et al*. 2014). Given continuous exposure to outside environment, how the skin cells resist oxidative stress is very important (Balic and Mokos 2019). Our study found that ACOT7, which may increase with the rising expression of iron load, has a protective effect on lipid peroxidation induced by H_2_O_2_, Erastin, and RSL3 in ESCs.

Previous studies showed that free iron induces the production of free radicals, contributing to an increase of the level of lipid peroxidation (Wuyun *et al*. 2019). The balance of intracellular iron homeostasis depends on the expression and activity of transferrin, ferritin, and other proteins, which regulate and store iron (Park and Chung 2019). When the amount of intracellular free iron is high, the expression of ferritin may store more excess iron (Shu *et al*. 2019). Autophagy also regulates intracellular free iron through ferritin degradation (Jacomin *et al*. 2019). Previous studies have shown that ACOT7 might regulate the metabolism of neuronal fatty acid and prevent neurotoxicity (Ellis *et al*. 2013). As ACOT7 is closely related to lipid metabolism, we discovered that ACOT7 might inhibit lipid peroxidation in ESCs. H_2_O_2_, Erastin, and RSL3 were applied to induce lipid peroxidation in ESCs because Erastin and RSL3 are classical inducers of ferroptosis (Shintoku *et al*. 2017). Erastin impairs cellular antioxidant defenses, facilitating toxic ROS accumulation by inhibiting system xc^−^, cysteine-dependent glutathione (GSH) synthesis, and inhibiting the trans-plasma membrane cysteine redox shuttle (Banjac *et al*. 2008; Dixon *et al*. 2012). RSL3 can directly decrease the expression of glutathione peroxidase 4 (GPX4), elevating the ROS level in cells (Sui *et al*. 2018).

We found that the overexpression of ACOT7 may decrease MDA and lipid peroxidation levels, resulting in a higher survival rate of ESCs. We also found that the overexpression of ACOT7 had no impact on cell proliferation and apoptosis, which suggests that ACOT7 may improve the cellular survival rate by inhibiting ferroptosis.

It is not clear how ACOT7 contributes to protecting ESCs against lipid peroxidation. Previous studies have shown that ACOT7 might interact with a series of long-chain acyl-CoA enzymes, especially arachidonoyl-CoA (AA-CoA), an important precursor of AA-phosphatidylethanolamine (AA-PE) that forms the cell membrane. Besides, AA-PE is likely to be attacked by oxidation, which is a signal of ferroptosis (Doll *et al*. 2017; Kagan *et al*. 2017). However, further research is needed to explore whether ACOT7 may regulate the level of AA-PE on the cell membrane to execute its function of inhibiting lipid peroxidation.

In conclusion, we found that ACOT7 may protect cells against lipid peroxidation and improve the cellular survival rate for the first time, which provides us with a new method to inhibit the damage of ESCs caused by lipid peroxidation under oxidative stress, a new idea to prevent ferroptosis and a new direction to explore how ACOT7 exercises its functions.

## Data Availability

Not applicable.

## Abbreviations

ESCs: Epidermal stem cells
ACOT7: Acyl-CoA thioesterase 7
MDA: Malondialdehyde
ROS: Reactive oxygen species
SPF: Specific pathogen-free
PBS: Phosphate-buffered saline
CK14: Cytokeratin 14
CK15: Cytokeratin 15
CK10: Cytokeratin 10
CCK-8: Cell Counting Kit-8
FTH: Ferritin Heavy Chain
FTL: Ferritin Light Chain
AA-CoA: Arachidonoyl-CoA
AA-PE: AA-phosphatidylethanolamine
